# Genetic Regulation of Dna2 Localization During the DNA Damage Response

**DOI:** 10.1534/g3.115.019208

**Published:** 2015-07-10

**Authors:** Askar Yimit, Michael Riffle, Grant W. Brown

**Affiliations:** * Department of Biochemistry and Donnelly Centre, University of Toronto, Toronto, ON M5S 3E1, Canada; † Department of Biochemistry, University of Washington, Seattle, Washington 98195

**Keywords:** Dna2 foci, DNA damage, double-strand breaks, DNA repair, colocalization, nuclear foci, genome stability, *Saccharomyces cerevisiae*, genetics

## Abstract

DNA damage response pathways are crucial for protecting genome stability in all eukaryotes. *Saccharomyces cerevisiae* Dna2 has both helicase and nuclease activities that are essential for Okazaki fragment maturation, and Dna2 is involved in long-range DNA end resection at double-strand breaks. Dna2 forms nuclear foci in response to DNA replication stress and to double-strand breaks. We find that Dna2-GFP focus formation occurs mainly during S phase in unperturbed cells. Dna2 colocalizes in nuclear foci with 25 DNA repair proteins that define recombination repair centers in response to phleomycin-induced DNA damage. To systematically identify genes that affect Dna2 focus formation, we crossed Dna2-GFP into 4293 nonessential gene deletion mutants and assessed Dna2-GFP nuclear focus formation after phleomycin treatment. We identified 37 gene deletions that affect Dna2-GFP focus formation, 12 with fewer foci and 25 with increased foci. Together these data comprise a useful resource for understanding Dna2 regulation in response to DNA damage.

The maintenance of the genome stability is an essential process in living cells. DNA lesions perturb cellular DNA replication and transcription processes, and failure to repair damaged DNA can lead to mutagenesis, tumorigenesis, and lethality. To combat DNA damage, cells activate DNA damage response mechanisms to arrest cell cycle progression, detect DNA lesions, amplify the DNA damage signal, and execute DNA repair (Rouse and Jackson 2002; Harrison and Haber 2006).

Dna2 is a conserved DNA-specific ATPase present in organisms from yeast to humans. Dna2 has both helicase and nuclease activities that are essential for Okazaki fragment maturation (Budd and Campbell 1997), and it plays a crucial role in repairing DNA double-strand breaks (Zhu *et al.* 2008) and in telomere and mitochondrial DNA maintenance (Choe *et al.* 2002; Duxin *et al.* 2009; Budd and Campbell 2013; Ronchi *et al.* 2013). Depletion of Dna2 causes incomplete DNA replication and genomic instability (Budd and Campbell 1995; Liu *et al.* 2000), and the ATPase and nuclease activities of Dna2 are essential for cell survival (Lee *et al.* 2000; Budd *et al.* 2000; Budd *et al.* 1995; Formosa and Nittis 1999). Overexpression of Dna2 has been detected in a variety of cancers and is associated with poor patient outcome (Strauss *et al.* 2014; Peng *et al.* 2012).

DNA double-strand breaks occur upon exposure to exogenous agents, such as ionizing radiation, or indirectly through replication fork collapse at DNA damage sites. If left unrepaired, double-strand breaks can cause genomic instability, cell death, and tumorigenesis (Mehta and Haber 2014; Jackson and Bartek 2009). Dna2 participates in DNA repair by homologous recombination (HR). In HR, Sae2 and the MRX (Mre11-Rad50-Xrs2) complex initiate DNA resection at the double-strand break, whereas long-range DNA resection is catalyzed either by Exo1 or by Dna2 in collaboration with the Sgs1/Top3/Rmi1 helicase-topoisomerase complex (Mimitou and Symington 2008; Zhu *et al.* 2008). The resulting 3′ single-stranded DNA is coated by Replication Protein A (RPA), which serves as a substrate for Rad51 filament formation (mediated by Rad52, Rad55-Rad57, and Rad54) and as a primer for subsequent DNA synthesis following strand invasion at a homologous DNA sequence that serves as a template for repair (Sugiyama and Kowalczykowski 2002; Sung 1997; Chen *et al.* 2013).

Dna2 has recently been shown to be a target of different post-translation regulation pathways. In fission yeast, Dna2 is phosphorylated by the checkpoint effector kinase Cds1 during replication stress, and phosphorylation is essential to stabilize stalled replication forks and to prevent reversal of arrested forks (Hu *et al.* 2012). In budding yeast, Dna2 is a direct target of Cdk1 and Mec1 kinases, and Dna2 itself directly stimulates Mec1 kinase activity (Chen *et al.* 2011; Kumar and Burgers 2013; Kosugi *et al.* 2009). Dna2 is also regulated by the SUMO pathway (Makhnevych *et al.* 2009). Previous studies indicate that Dna2 forms nuclear foci during DNA damage and DNA replication stress (Lisby and Rothstein 2009; Makhnevych *et al.* 2009; Tkach *et al.* 2012). Here we characterize the formation of Dna2 foci in response to double-strand DNA breaks and apply a genome-wide screen to systematically identify gene deletion mutants that change Dna2 focus formation levels.

## Materials and Methods

### Strains and media

Strains used in this study are listed in Supporting Information, Table S1 and are derivatives of BY4741 (Brachmann *et al.* 1998). Low-fluorescence media [yeast nitrogen base supplemented with 5 g/l ammonium sulfate, 2% (w/v) glucose, 150 mg/l methionine, 20 mg/l histidine, 100 mg/l leucine, and 20 mg/l uracil] was used for high-throughput screening.

The *DNA2-yEmCherry* strain was constructed by transforming JTY5 with a PCR product containing *yEmCherry*::*CaURA3* and targeted to DNA2 (Dna2-mOrr-fw and Dna2-mOrr-rv primers; Table S2). The template for the PCR, pKT-yEmCherry-CaURA3, was constructed by replacing mCherry sequences in the plasmid pKT-mCherry-CaURA3 with yEmCherry sequences amplified from pNEB31 (Silva *et al.* 2012) with primers yEmRFP_F and yEmRFP_R (Table S2). The plasmid pKT-mCherry-CaURA3 was constructed by replacing GFP in the plasmid pKT209 (Sheff and Thorn 2004) with mCherry, and was a kind gift from Mike Cox in Brenda Andrews’ laboratory.

### Microscopy and image analysis

For analysis of Dna2-GFP nuclear foci, GFP fusion proteins that colocalized with Dna2-yEmCherry, and Dna2-GFP foci in gene deletion backgrounds, cultures were grown to saturation in YPD, diluted into fresh YPD to OD_600_ = 0.1, and grown for 2 hr at 30° before treating with 5 µg/ml phleomycin for 2 hr. Eleven z slices with a 0.4 µm step size were acquired using Volocity imaging software (PerkinElmer) controlling a Leica DMI6000 microscope with the fluorescein isothiocyanate, Texas Red, and differential interference contrast filter sets (Quorum Technologies). Dna2-yEmCherry foci, ORF-GFP foci, and colocalizing foci were counted in at least 100 cells. Functions of the proteins tested for colocalization with Dna2 were annotated with GO-Slim terms downloaded from the Saccharomyces Genome Database (www.yeastgenome.org; accessed on 4 April 2015) and GO functions from GeneMANIA (www.genemania.org; accessed on 3 April 2015) (Montojo *et al.* 2014). Protein interactions for the proteins tested for Dna2 colocalization were downloaded from GeneMANIA using data from BioGRID (www.thebiogrid.org) small-scale studies.

### Identification of Dna2 focus regulators

*DNA2-GFP* (AYY3) was crossed with an array of 4293 strains (Costanzo *et al.* 2010) from the haploid nonessential yeast gene deletion collection (Giaever *et al.* 2002) using synthetic genetic array methodology (Baryshnikova *et al.* 2010). The resulting strains, expressing Dna2-GFP in the context of deletion of individual nonessential genes, were grown and imaged after treatment with phleomycin or with vehicle as a control, as described previously (Tkach *et al.* 2012). Briefly, the haploid strains were grown to saturation overnight in minimal media and further sub-cultured to mid-log phase (∼16 hr growth time) in low fluorescence media. Cells were transferred to 384-well slide plates to a final density of 0.045 OD_600_ ml^−1^ and incubated at 30° for 2 hr in low fluorescence medium (control) or low fluorescence medium plus 5 µg/ml phleomycin. Images from four fields per well were acquired in the green (405/488/640 primary dichroic, 540/75 emission band-pass filter, 800 ms exposure) and red channels (405/561/640 primary dichroic, 600/40 emission band-pass filter, 2000 ms exposure) on an EVOTEC Opera confocal microscope system (PerkinElmer). The complete set of images from the high-throughput screen is available from the Yeast Resource Center Public Image Repository (Riffle and Davis 2010) at http://images.yeastrc.org/yimit-2015. The images were scored by visual inspection for strains that exhibited decreases in Dna2-GFP foci in phleomycin or increases in Dna2-GFP foci in the untreated samples, relative to control. Positives were examined in low throughput as indicated above. The number of Dna2-GFP foci per cell was quantified by visual analysis of at least 100 cells, in duplicate. We assessed whether the mean number of Dna2-GFP foci per cell in each mutant was detectably different from wild-type by applying a two-tailed *t*-test, assuming equal variance. The network of genes that affect Dna2 focus formation was drawn in Cytoscape (www.cytoscape.org) and overlaps with other data sets were assessed using a hypergeometric test in R. GO term enrichment was analyzed with the GO Term Finder (go.princeton.edu) using the deletion collection screened as the "universe," and p-values corrected for multiple testing are reported.

For gene deletions with a decreased fraction of cells with Dna2-GFP foci, the total GFP fluorescence and nuclear GFP fluorescence were measured after segmenting 10 cells and nuclei for each mutant in ImageJ (http://imagej.nih.gov/ij/). Nuclear focus intensity was measured by segmenting 15–20 individual foci and measuring the GFP fluorescence in ImageJ. Unbudded (G1) cells were excluded from the analysis. We assessed whether the mean GFP fluorescence intensity per cell, the mean nuclear GFP fluorescence intensity per cell, and the mean nuclear focus GFP fluorescence intensity in each mutant were detectably different from wild-type by applying a two-tailed *t*-test, assuming equal variance.

### Drug sensitivity:

To assay phleomycin sensitivity, cultures were grown overnight at 30° in YPD. Cultures were diluted to an OD_600_ of 1, serially diluted 10-fold, spotted on YPD medium with or without 2.5 or 5 µg/ml phleomycin, and grown for 2–3 d at 30° before imaging.

### Data availability

Strains are available upon request. The complete set of images from the Dna2-GFP focus screen is available from the Yeast Resource Center Public Image Repository at http://images.yeastrc.org/yimit-2015.

## Results and Discussion

### Dna2 forms nuclear foci in S and G2 phases

**Figure 1:**
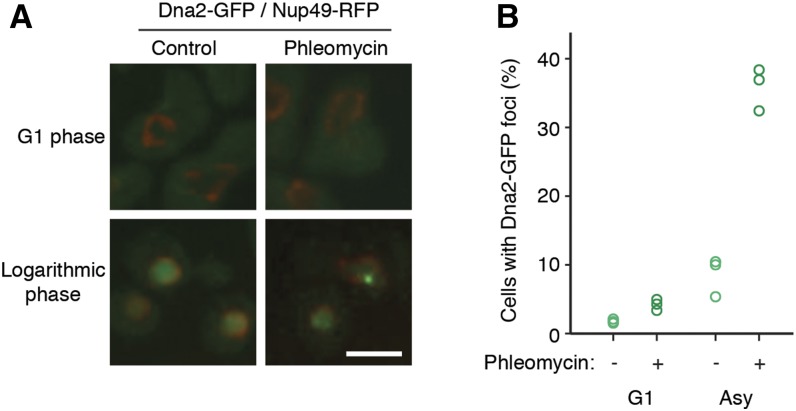
Dna2-GFP focus formation in G1 and asynchronous cells. **(**A) Logarithmic phase asynchronous cells or cells arrested in G1 were exposed to phleomycin (5 μg/ml) and imaged by confocal microscopy to detect Dna2-GFP foci. (B) The number of Dna2-GFP foci per cell was quantified for the G1-arrested and asynchronous cells. At least 100 cells were analyzed in three independent experiments, and the percent of cells with at least one Dna2-GFP focus is plotted for each.

Dna2, like many DNA damage response proteins, forms nuclear foci in response to double-strand breaks and DNA replication stress (Lisby and Rothstein 2009; Makhnevych *et al.* 2009; Tkach *et al.* 2012; Chen *et al.* 2011). In addition to being regulated by DNA damage, the intracellular localization of Dna2 is connected to cell cycle phase via CDK phosphorylation. In G1 arrested cells Dna2 is mainly cytoplasmic, whereas during S, G2, and M phases Dna2 displays a nuclear localization (Kosugi *et al.* 2009). To investigate the cell cycle distribution of Dna2 foci in unperturbed cells and cells with double-strand DNA breaks, we quantified Dna2-GFP foci in unbudded (G1), small budded (S), and large budded (G2) cells in both asynchronous cultures and cultures treated with phleomycin. Phleomycin, an antibiotic of the bleomycin family, causes free radical–mediated DNA damage, including double-strand breaks (Moore 1988; Sleigh 1976). In unperturbed cells, Dna2 foci were mainly found in S phase in 24% of small budded cells (Table 1), suggesting that Dna2 foci can arise during DNA replication. Following 2 hr of treatment with phleomycin, Dna2 foci were found in small budded and large budded cells, but rarely in unbudded (G1) cells (Table 1). We arrested cells in G1 with mating pheromone and treated the arrested cells with phleomycin (Figure 1, A and B), confirming that Dna2 foci do not form efficiently during G1 phase. These results are in agreement with the established roles of Dna2 in Okazaki fragment maturation (in S phase) and roles in double-strand break repair (DNA resection during G2/M phase).

**Table 1:** Frequencies of Dna2-GFP focus formation in G_1_, S, and G_2_/M cells

Unbudded (G_1_)		Small Budded (S)		Large Budded (G_2_/M)	
Control	Phleomycin	Control	Phleomycin	Control	Phleomycin
1.7	0	24.2	53.1	9	63.8

The percent of cells in each morphological class containing a Dna2-GFP focus is indicated.

### Dna2 colocalizes with proteins that form Rad52 repair centers at double-strand breaks

**Figure 2:**
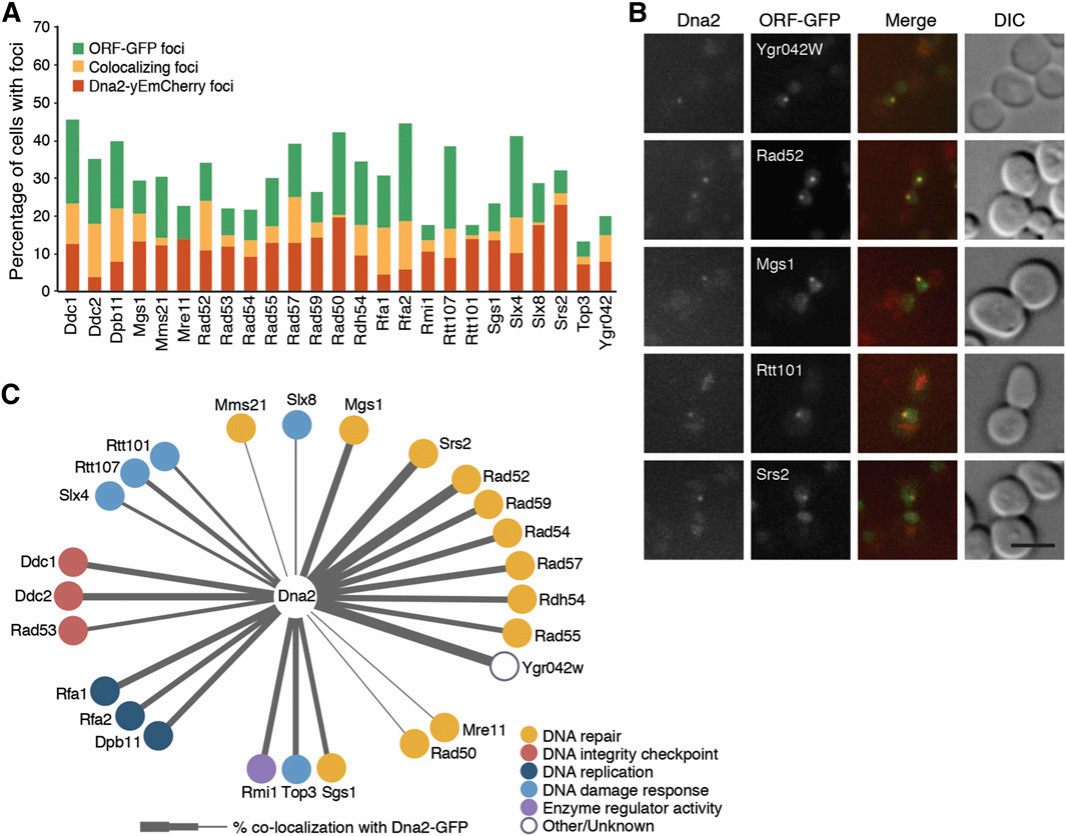
Colocalization of Dna2 with DNA repair and DNA damage response proteins. **(**A) Colocalization of the indicated ORF-GFP with Dna2-yEmCherry was measured by confocal fluorescence microscopy after phleomycin treatment. The percent of cells with colocalizing foci is plotted. (B) Representative images of proteins that colocalize with Dna2. Cells expressing Dna2-yEmCherry and the indicated ORF-GFPs were imaged after phleomycin treatment. Scale bar is 5 μm. (C) Network of the 25 proteins that colocalize with Dna2 after phleomycin treatment. Thickness of the edges corresponds to the fraction of cells displaying colocalization. Gene function is indicated by node color.

In response to double-strand breaks, a number of DNA repair and checkpoint proteins relocalize from diffuse nuclear distribution to distinct sub-nuclear foci. The recombination repair protein Rad52 forms foci that colocalize with double-strand breaks (Lisby *et al.* 2003; Lisby *et al.* 2001), and some repair proteins are known to colocalize in foci with Rad52 (Lisby *et al.* 2004). Not all proteins that form nuclear foci colocalize with Rad52, however (Tkach *et al.* 2012; Gallina *et al.* 2015). To systematically analyze proteins that colocalize with Dna2 in response to phleomycin, we fused Dna2 to yeast-enhanced monomeric Cherry (yEmCherry) (Keppler-Ross *et al.* 2008; Silva *et al.* 2012) and crossed it to 55 GFP-tagged ORF strains, including 27 proteins that we found to form nuclear foci in DNA replication stress (Tkach *et al.* 2012) and an additional 28 proteins reported to form nuclear foci in DNA damage (Gasior *et al.* 1998; Melo *et al.* 2001; Frei and Gasser 2000; Lisby *et al.* 2004; Srikumar *et al.* 2013; Denervaud *et al.* 2013). Among the 55 proteins, 25 colocalize with Dna2 detectably, with the extent of colocalization ranging from 55% (Ygr042w) to 2% (Mre11) (Table 2, Figure 2, and Table S3).

**Table 2:** Proteins that colocalize with Dna2-yEmCherry during treatment with phleomycin

ORF-GFP	Protein	Protein-GFP Colocalized with Dna2-yEmCherry (%)	Dna2-yEmCherry Colocalized with Protein-GFP (%)
YGR042W	Ygr042w	55	46
YML032C	Rad52	54	51
YJL090C	Dpb11	41	63
YDR499W	Ddc2	40	76
YNL218W	Mgs1	38	36
YAR007C	Rfa1	37.5	71
YPL024W	Rmi1	37.5	18.7
YDR004W	Rad57	36.7	48.6
YLR234W	Top3	36	21
YDL059C	Rad59	32	21
YGL163C	Rad54	30	30
YPL194W	Ddc1	29	45.4
YBR073W	Rdh54	28.5	42.8
YJL092W	Srs2	28.5	9.5
YPL153C	Rad53	28.2	20
YJL047C	Rtt101	28	7
YNL312W	Rfa2	26.2	64
YLR135W	Slx4	26	46
YHR154W	Rtt107	22.4	46.1
YMR190C	Sgs1	22	15.3
YDR076W	Rad55	20.8	22
YEL091C	Mms21	11	14.2
YER116C	Slx8	5.8	3.3
YNL250W	Rad50	3	3.2
YMR224C	Mre11	2.1	1.4

We plotted a network of the proteins that colocalize detectably with Dna2 foci (Figure 2C), with edge thickness corresponding to the percent of foci that colocalize with Dna2 foci, and with Gene Ontology process term indicated for each. Consistent with the roles of Dna2 in DNA replication and double-strand break repair, we found that most of the 25 proteins that colocalize with Dna2 in phleomycin have connections to repair of double-strand breaks. All the representatives of the *RAD52* epistasis group that were tested colocalized with Dna2, with Rad52 found with Dna2 at the highest frequency (54% of Dna2 foci contain Rad52). Of particular interest, Ygr042w showed a similarly high frequency of colocalization with Dna2, consistent with a recent report that the fission yeast homolog, Dbl2, colocalizes with recombination repair foci (Yu *et al.* 2013), suggesting that Ygr042w could play a role in recombination repair. Dna2 functions in the resection step of double-strand break repair in concert with Sgs1/Top3/Rmi1 (Zhu *et al.* 2008) and colocalized frequently with each of the members of the complex (Figure 2C). Dna2 also colocalized frequently with the ssDNA binding protein RPA, a regulator of resection (Niu *et al.* 2010; Cejka *et al.* 2010; Chen *et al.* 2013). Dna2 showed only a weak colocalization with the MRX complex (Mre11, Rad50, Xrs2), consistent with two-step resection models in which ends resected initially by MRX/Sae2 are handed off to Dna2 (Mimitou and Symington 2011). As previously suggested for MRX and Rad52 (Lisby *et al.* 2004), the weak colocalization of Dna2 and MRX detected could reflect proteins that are in the same repair center but that are not associated with the same DNA end, because multiple DNA ends can associate with a single Rad52 focus (Lisby *et al.* 2003). DNA damage checkpoint proteins colocalize with Dna2 robustly, including the Mec1 activators Dpb11, Ddc1, and Ddc2, and the effector kinase Rad53 (Figure 2C). We found that complex members tended to show similar frequencies of colocalization (Rfa1/Rfa2, Sgs1/Top3/Rmi1, MRX, RFC, Rtt107/Slx4, Rad55/57) (Figure 2C and Table S3).

**Figure 3:**
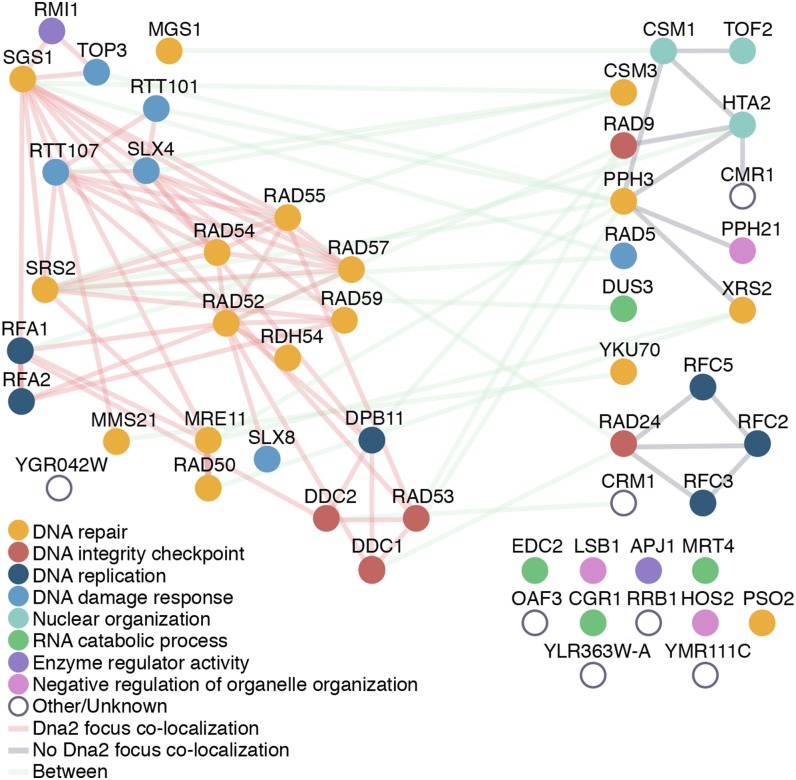
Dna2 focus protein–protein interaction network. The proteins that were tested for Dna2 focus colocalization are represented as nodes colored according to function, with the 25 proteins detected at the Dna2 focus on the left and the 28 proteins not found in Dna2 foci on the right. Edges correspond to protein–protein interactions curated by BioGRID, with interactions between Dna2 focus proteins in red, interactions between non-Dna2 focus proteins in blue, and interactions that bridge the two groups in green.

We analyzed the extent of protein–protein interactions among the 25 proteins that colocalize with Dna2 foci, and among the 28 proteins that form nuclear foci but do not colocalize detectably with Dna2 foci following phleomycin treatment (Figure 3). We noted that the proteins that colocalized with Dna2 form a dense network of protein–protein interactions (5.04 interactions on average), whereas the protein–protein interactions among the proteins that did not colocalize with Dna2 are sparser (1.75 interactions on average). Additionally, proteins that colocalize with Dna2 foci for the most part are annotated on GO processes involved in DNA repair, DNA replication, and DNA damage response. The proteins that did not colocalize with Dna2 foci are involved in some distinct processes, notably RNA catabolism, suggesting that some of these proteins form nuclear foci with functions that are distinct from Rad52 repair centers. Consistent with this possibility, Cmr1 was recently shown to form a distinct intranuclear compartment that also contains four additional proteins that fail to colocalize with Dna2 (Pph3, Apj1, Hos2, and Dus3) (Gallina *et al.* 2015). Together our data indicate that Dna2 foci colocalize with a subset of repair and checkpoint proteins that likely define the canonical Rad52 double-strand break repair foci (Gasior *et al.* 1998; Lisby *et al.* 2001, 2004; Yu *et al.* 2013).

### Identification of genes affecting Dna2 focus formation

**Figure 4:**
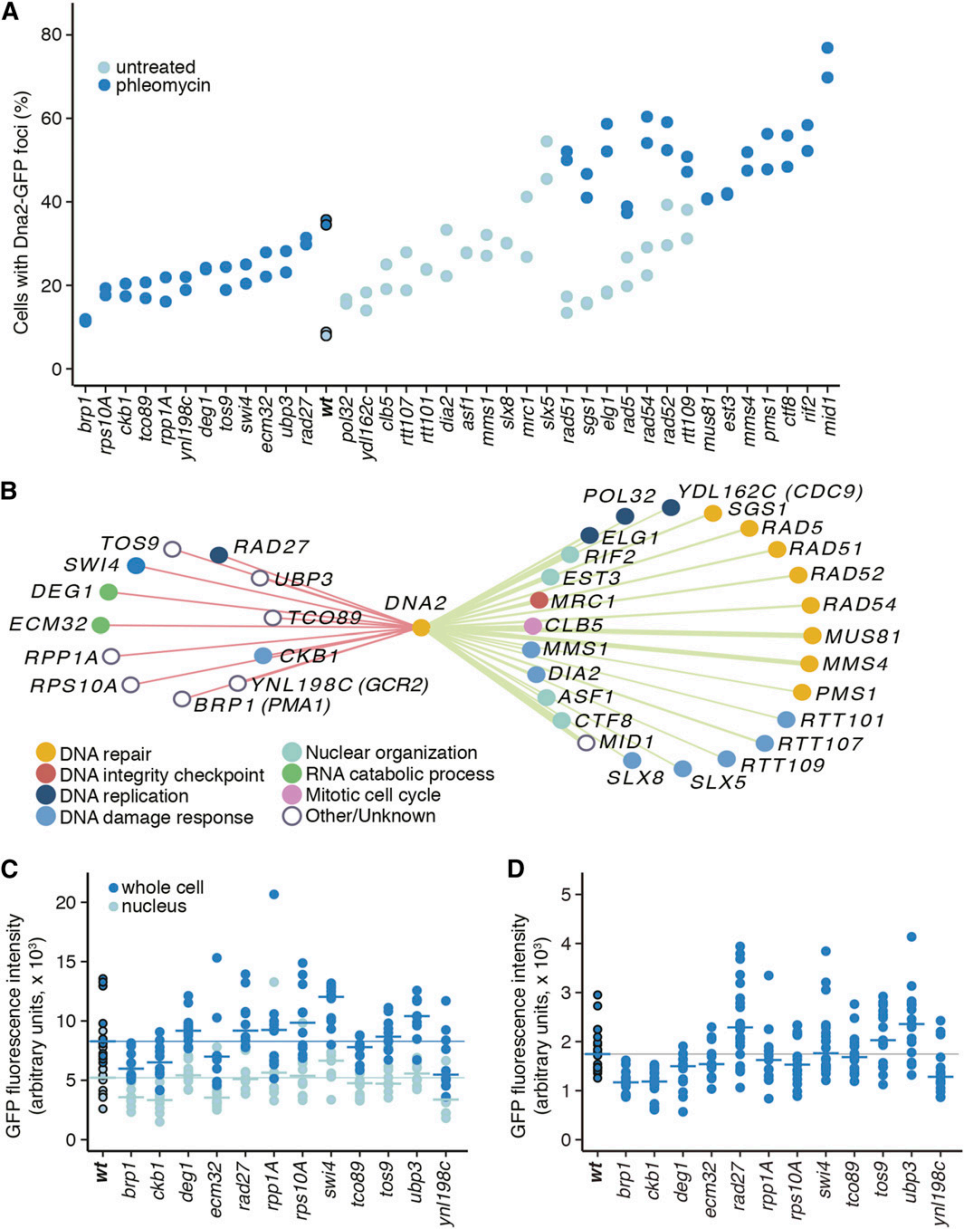
Identification of genes that affect Dna2-GFP focus formation. (A) The fraction of cells with Dna2-GFP foci in 12 gene deletions with fewer Dna2 foci after treatment with phleomycin and in 25 gene deletions with increased Dna2 foci either in untreated cells or after treatment with phleomycin is plotted for two replicates. (B) The 37 confirmed genes that affect Dna2 foci abundance are organized in a network with nodes colored according to function. Edges are in red for gene deletions with fewer foci and in green for gene deletions with increased foci. (C) Dna2-GFP fluorescence intensity after treatment with phleomycin is plotted for whole cells (dark blue; n = 10) and nuclei (light blue; n = 10). Horizontal bars indicate the median Dna2-GFP fluorescence in each compartment for each mutant. Horizontal lines mark the median Dna2-GFP fluorescence in each compartment for wild-type. (D) Dna2-GFP nuclear focus intensity after treatment with phleomycin is plotted for each mutant (n = 12 to 20) and wild-type. Horizontal bars indicate the median focus intensity for each mutant, and the horizontal line marks the median focus intensity for wild-type.

To systematically identify the genetic requirements for Dna2 focus formation, we screened a collection of 4293 haploid nonessential gene deletion mutants (Costanzo *et al.* 2010; Giaever *et al.* 2002) in the absence and presence of phleomycin. Dna2-GFP foci were visualized by high-throughput confocal microscopy and scored by visual inspection. All images from the screen are available from the Yeast Resource Center Public Image Repository (Riffle and Davis 2010) at http://images.yeastrc.org/yimit-2015. Forty-seven genes were identified that affected Dna2-GFP focus formation, either by increasing focus formation in untreated cells (32 genes) or by decreasing focus formation in phleomycin-treated cells (15 genes) (Table S4). These positives were reimaged in low throughput before and after treatment with phleomycin for 2 hr, and foci in the resulting images were quantified. We confirmed that 12 mutants showed a decrease (*P* < 0.05) in the fraction of cells with a Dna2-GFP focus following phleomycin treatment, relative to wild-type (Figure 4A), and that 25 mutants had an increased (*P* < 0.05) fraction of cells with Dna2 foci relative to wild-type (Figure 4A). We identified three classes of mutants with increased Dna2 foci: those with increased spontaneous foci only (11), those with increased spontaneous and increased phleomycin-induced foci (7), and those with increased phleomycin-induced foci only (7) (Figure 4A). There are likely additional mutants in the deletion collection that cause increased Dna2 foci in phleomycin only, because this class was not scored in our primary screen.

The 25 gene deletions that conferred increased Dna2 foci were strongly enriched for genes involved in DNA repair and DNA damage response (Figure 4B) (*P* = 2×10^−17^ and *P* = 5×10^−16^). We compared these genes to those identified in a recent "constitutive *RNR3* expression" screen (Hendry *et al.* 2015) and found significant overlap (16 genes, hypergeometric *P* = 4×10^−21^), suggesting the presence of increased spontaneous DNA damage in these mutants, as expression of *RNR3* responds specifically to DNA damage (Elledge and Davis 1990). We compared the genes that, when deleted, caused increased Dna2 foci to those that cause increased Rad52 foci (Alvaro *et al.* 2007), again finding significant overlap (10 genes, hypergeometric *P* = 2×10^−11^). Finally, we compared the set of genes with negative genetic interactions with *dna2-1* or *dna2-2* (Budd *et al.* 2005), which could indicate spontaneous damage that requires Dna2 for its repair. We noted a significant overlap (10 genes, hypergeometric *P* = 2×10^−14^). There were only five genes (*CLB5*, *CTF8*, *EST3*, *MID1*, and *RIF2*) in our set of 25 that were not found to have increased Rnr3 expression, increased Rad52 foci, or a negative genetic interaction with *dna2*. Decreased replication origin usage in *clb5* mutants is proposed to cause spontaneous DNA damage (Gibson *et al.* 2004). Deletion of the *CTF18* gene, which encodes the binding partner of Ctf8, causes increased Rad52 foci (Gellon *et al.* 2011). *RIF2* and *EST3* regulate telomere length (Wotton and Shore 1997; Hughes *et al.* 2000). *MID1* has no clear connection to DNA repair, but it is only 403 bp from the 5′ end of the *RFC3* ORF. *RFC3* is essential for DNA replication and is important for DNA repair (Cullmann *et al.* 1995; Green *et al.* 2000). Thus, all 25 of the genes whose deletion causes increased Dna2 focus formation likely cause increased DNA damage when deleted, and Dna2 likely participates in the repair of that damage.

The 12 gene deletions with fewer Dna2-GFP foci do not represent a coherent functional group (Figure 4B) and were not enriched for any GO term. Of the genes identified, only *RAD27* has a clear connection to Dna2, because Rad27 and Dna2 function in concert in Okazaki fragment maturation (Ayyagari *et al.* 2003). Several of the genes we identify are involved in translation capacity (*RPP1A*, *RPS10A*, *DEG1*), nutrient sensing (*TCO89*), and G1 transit (*SWI4*), and therefore could reflect cell cycle delays in G1, where Dna2 foci typically do not form (Table 1). It is also possible that reduced Dna2-GFP foci per cell could be caused by decreased total abundance of Dna2-GFP or decreased nuclear abundance of Dna2-GFP. We tested these latter possibilities by quantifying total GFP fluorescence per cell, nuclear GFP fluorescence per cell, and Dna2-GFP focus intensity (Figure 4, C and D, and Table S4). Two mutants, *brp1*∆ and *ckb1*∆, had statistically apparent decreases in nuclear Dna2-GFP mean fluorescence intensity (Figure 4C). In both cases, the effect size was small (0.67× wild-type for *brp1*∆ and 0.70× wild-type for *ckb1*∆), and the decrease in nuclear Dna2-GFP signal was paralleled by a similar decrease in total cellular Dna2-GFP signal. Deletion of *YNL198C* caused a decrease in total cellular Dna2-GFP fluorescence, but the decrease in nuclear fluorescence in this mutant could not be statistically distinguished from wild-type. A similar analysis of Dna2-GFP focus intensity in the 12 mutants with decreased Dna2-GFP foci per cell revealed four strains with decreased focus intensity (Figure 4D) (*brp1*∆, *ckb1*∆, *deg1*∆, and *ynl198c*∆). Interestingly, two mutants, *rad27*∆ and *ubp3*∆, had increased focus intensity despite having fewer foci per cell. We conclude that none of the mutants causes a substantial decrease in Dna2-GFP expression or in nuclear abundance of Dna2-GFP. However, in *brp1*∆ and *ckb1*∆, decreased nuclear localization could indirectly cause a decrease in Dna2-GFP focus intensity.

**Figure 5:**
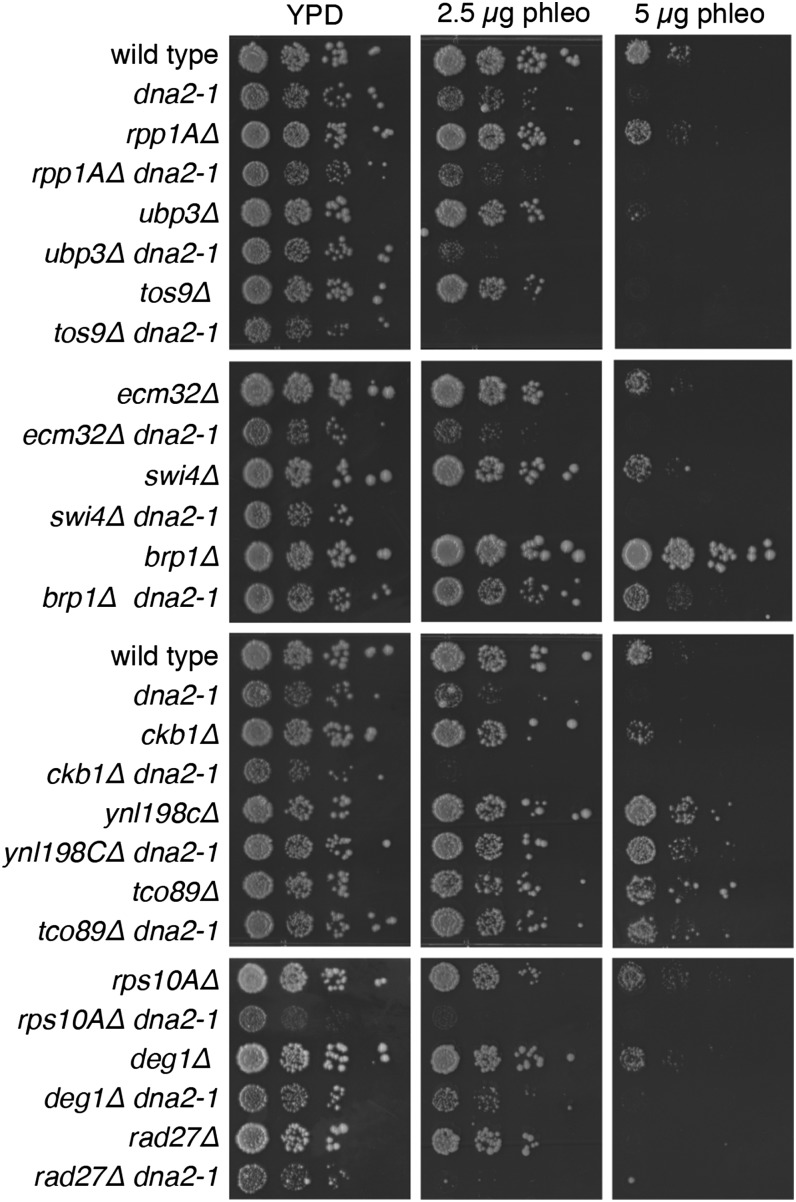
Phleomycin sensitivity of *dna2-1* double mutants. Ten-fold serial dilutions of the indicated strains were spotted onto YPD or YPD containing phleomycin at the concentrations shown. Plates were imaged after 2–3 d.

To further assess the functional relationship between *DNA2* and gene deletions that decrease Dna2-GFP foci, single and double mutants of *dna2-1* (Budd *et al.* 2000) and *ubp3Δ*, *ecm32*Δ, *swi4Δ*, *ckb1Δ*, *ynl198CΔ*, *deg1Δ*, *rad27Δ*, *rpp1A∆*, *tos9∆*, *brp1∆*, *tco89∆*, and *rps10A∆* were tested for phleomycin sensitivity. Double mutants of *dna2-1* with *tos9∆*, *swi4Δ*, *ckb1Δ*, *rps10A∆*, or *rad27Δ* showed increased phleomycin sensitivity relative to the relevant single mutants (Figure 5), indicating that deletion of any of these five genes exacerbates the phleomycin sensitivity of *dna2-1* mutants.

In summary, we find that Dna2 nuclear foci induced by phleomycin colocalize with a group of proteins that form double-strand break repair centers with Rad52. We identified 25 genes that cause an increase in Dna2 foci when mutant, likely by promoting spontaneous DNA damage. We identified a functionally diverse group of 12 genes that are important for robust Dna2 focus formation in phleomycin, five of which contribute to phleomycin resistance. Together these data will be a useful resource for understanding Dna2 compartmentalization in response to DNA damage.

## Supplementary Material

Supporting Information
