# Supplementary material for: Genetic Regulation of Dna2 Localization During the DNA Damage Response
Source: G3 (Bethesda). 2015 Jul 10;5(9):1937–44. doi: 10.1534/g3.115.019208 (PMC4555230; doi:10.1534/g3.115.019208)
Supplement: Supporting Information [file supp_5_9_1937__index.html]

Genetic Regulation of Dna2 Localization During the DNA Damage Response — Supporting Information 

# Genetic Regulation of Dna2 Localization During the DNA Damage Response

## Supporting Information for Yimit, Riffle, and Brown, 2015

**Files in this Data Supplement:**

- Table S1 - Strains used in this study. (.xlsx, 9 KB)
- Table S2 - Primers used in this study. (.xlsx, 8 KB)
- Table S3 - GFP fusion proteins that co-localize with Dna2 during phleomycin treatment. (.xlsx, 10 KB)
- Table S4 - Gene deletion mutants that affect Dna2-GFP foci formation. (.xlsx, 11 KB)
